# Illuminating the “Black Box” of Progesterone-Dependent Embryo Implantation Using Engineered Mice

**DOI:** 10.3389/fcell.2021.640907

**Published:** 2021-04-07

**Authors:** Vineet K. Maurya, Francesco J. DeMayo, John P. Lydon

**Affiliations:** ^1^Department of Molecular and Cellular Biology, Baylor College of Medicine, One Baylor Plaza, Houston, TX, United States; ^2^Reproductive and Developmental Biology Laboratory, National Institute of Environmental Health Sciences, Durham, NC, United States

**Keywords:** progesterone, endometrium, implantation, receptivity, decidualization, human, mouse, mediators and modifiers

## Abstract

Synchrony between progesterone-driven endometrial receptivity and the arrival of a euploid blastocyst is essential for embryo implantation, a prerequisite event in the establishment of a successful pregnancy. Advancement of embryo implantation within the uterus also requires stromal fibroblasts of the endometrium to transform into epithelioid decidual cells, a progesterone-dependent cellular transformation process termed decidualization. Although progesterone is indispensable for these cellular processes, the molecular underpinnings are not fully understood. Because human studies are restricted, much of our fundamental understanding of progesterone signaling in endometrial periimplantation biology comes from *in vitro* and *in vivo* experimental systems. In this review, we focus on the tremendous progress attained with the use of engineered mouse models together with high throughput genome-scale analysis in disclosing key signals, pathways and networks that are required for normal endometrial responses to progesterone during the periimplantation period. Many molecular mediators and modifiers of the progesterone response are implicated in cross talk signaling between epithelial and stromal cells of the endometrium, an intercellular communication system that is critical for the ordered spatiotemporal control of embryo invasion within the maternal compartment. Accordingly, derailment of these signaling systems is causally linked with infertility, early embryo miscarriage and gestational complications that symptomatically manifest later in pregnancy. Such aberrant progesterone molecular responses also contribute to endometrial pathologies such as endometriosis, endometrial hyperplasia and cancer. Therefore, our review makes the case that further identification and functional analysis of key molecular mediators and modifiers of the endometrial response to progesterone will not only provide much-needed molecular insight into the early endometrial cellular changes that promote pregnancy establishment but lend credible hope for the development of more effective mechanism-based molecular diagnostics and precision therapies in the clinical management of female infertility, subfertility and a subset of gynecological morbidities.

## Introduction

Estimated to reach eight billion by 2024 (United Nations, 2019), the projected size of the global human population is impressive. However, this population number will be attained not because of human fecundity but despite it. Seminal studies by Wilcox and others revealed that the chance for a live birth per natural menstrual cycle [the monthly fecundity rate (MFR)] is only ∼30% for healthy couples in their reproductive prime (Zinaman et al., 1996; Wilcox et al., 1999; Macklon et al., 2002). The remarkably low human MFR stands in stark contrast to other primate species with MFRs reaching ∼80%; reviewed in Macklon and Brosens (2014). Contributing to the low MFR in humans is that nearly 55% of conceptions terminate due to either implantation failure or preclinical miscarriage (Wilcox et al., 1999; Macklon et al., 2002). Because these pregnancy losses occur before the time of a missed menstrual period or before the rise in detectable embryonic-derived human chorionic gonadotropin (hCG), couples are usually unaware of the loss. In addition to preclinical losses, ∼10–15% of clinically recognized pregnancies miscarry within the first trimester (Chard, 1991; Macklon et al., 2002); the etiologic origin of many of these losses is thought to arise during the preclinical period (Norwitz et al., 2001).

The relatively low MFR in humans also sets limits for success when using assisted reproductive technology (ART), which includes *in vitro* fertilization followed by embryo transfer (IVF-ET); reviewed in Bischof et al. (2006) and Blesa et al. (2014). Current ART services commonly depend on the transfer of a morphologically high-grade embryo into a receptive endometrium to achieve a successful singleton pregnancy. Notwithstanding the extraordinary progress in ART over the past four decades, only ∼50% of embryos implant after transfer; of these, a mere ∼50% advance to live births (Boomsma et al., 2009; Kupka et al., 2014). These losses are distressing to patients and their fertility specialists alike, particularly when critical milestones for success (i.e., embryo number and quality) during an IVF-ET cycle have been reached.

Apart from deficiencies in embryo quality, defects in endometrial function are now considered important etiologic factors in implantation failure and early miscarriage following a natural or assisted conception (Valbuena et al., 2017; Valdes et al., 2017). Moreover, in the diagnosis of the underlying cause(s) of recurrent pregnancy loss (Rai and Regan, 2006; Bellver and Simon, 2018), a dysfunctional endometrium is frequently suspected as a causal factor after embryonic chromosomal abnormalities and maternal factors—endocrine, genetic, immunological, and thrombophilic disorders along with uterine anatomic defects—are eliminated as etiologic contributors.

Therefore, if we are to increase pregnancy success rates currently achieved by natural or assisted conception as well as improve outcomes for those women at high-risk for early pregnancy loss, significant expansion not only in our cellular but also molecular understanding of endometrial function during the periimplantation period is required. With the aforementioned as background, this review will profile a selection of pivotal molecular mechanisms, primarily revealed by mouse studies in conjunction with *in vitro* approaches, which are indispensable for progesterone-dependent endometrial receptivity and decidualization, cellular processes that ensure successful embryo implantation in members of the eutherian mammalian class, which includes the human and mouse.

## Human and Mouse: Different But the Same

Although comparative phylogenomics estimate that the human and murine lineages diverged ∼70–90 million years ago; reviewed in Nei and Glazko (2002), the mouse has proven to be an essential proxy for modeling human endometrial function *in vivo*. Despite being a distant evolutionary relative, the mouse shares with the human many important endometrial cellular responses to hormones during early gestation. While the initial cellular events of embryo implantation in the human endometrium are interstitial (i.e., the blastocyst fully embeds within the endometrium) as opposed to eccentric (i.e., the blastocyst implants within a uterine crypt) in the mouse (Cha et al., 2012b; Ander et al., 2019), the basic cellular responses to progesterone that prime the endometrium to be receptive to embryo implantation are common in both species. Therefore, these observations argue that the fundamental molecular mechanisms driving progesterone control of these endometrial cellular responses are also conserved, a consensus that has popularized the mouse as the “go-to” *in vivo* experimental surrogate for the human when illuminating the “black-box” of embryo implantation (Macklon et al., 2002).

Apart from the established arsenal of surgical and hormone treatment protocols specifically tailored for mouse periimplantation investigations, the genetic tractability of the mouse further underscores its versatility when assigning genes, pathways, and networks with a specific endometrial cellular response(s) at the whole organism level. Specifically, mouse engineering in general and conditional genetic technology in particular have proven invaluable interrogative methodologies in accelerating our functional understanding of progesterone-dependent endometrial cellular processes during the periimplantation window. However, a mouse study is not complete without follow-up translational validation, usually with *in vitro* and/or *ex vivo* systems that model the human [i.e., histological and/or cell analysis of human tissue, endometrial cell culture, co-culture or an organoid culture system (Fitzgerald et al., 2020)].

Before proceeding to the review’s main focus, an overview of the salient stages of the implantation process in the murine endometrium—with relevant cross comparisons to the human—is instructive to provide physiological and cellular context to the described associated progesterone-dependent molecular mechanisms that follow.

## Periimplantation Biology of the Murine Endometrium

### Synchronicity Underlies Embryo Implantation Success

**FIGURE 1:**
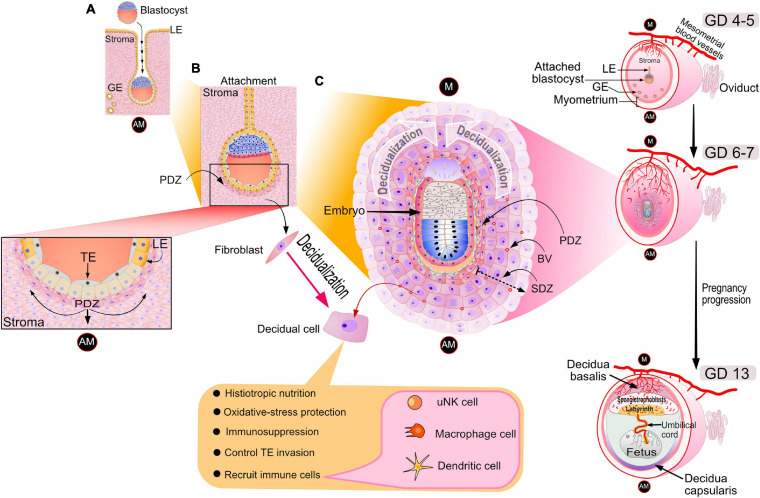
Cellular changes in the murine uterus during the periimplantation period. **(A)** On the evening of GD 4, the late-stage blastocyst attaches to the luminal epithelium of the implantation chamber (also known as the crypt) of the receptive endometrium. Note: GD 1 is defined here as the day an early morning vaginal plug is detected following overnight housing of the female with the male. The acronyms: LE, GE, AM denote luminal epithelium, glandular epithelium and antimesometrial pole respectively. **(B)** Following embryo attachment late on GD 4, subepithelial stromal cells surrounding the nidating blastocyst undergo extensive proliferation by the morning of GD 5. On the afternoon of GD 5, proliferating stromal fibroblasts surrounding the blastocyst differentiate to form an avascular primary decidual zone (PDZ), which initially expands toward the antimesometrial (AM) pole (see inset). At this time, the LE starts to degenerate (see inset); TE denotes the mural trophectoderm of the blastocyst, which soon breaches the LE divide to invade the underlying stroma. **(C)** By GD 6, the PDZ is well established, the implantation chamber epithelium is removed, and the formation of the secondary decidual zone (SDZ) surrounding the PDZ has occurred. At this time, cell proliferation is significantly decreased in the PDZ but continues in the SDZ, which expands and spreads to form the antimesometrial decidua toward the AM pole and subsequently the mesometrial decidua toward the M pole (the presumptive site for placentation). Containing terminally differentiated decidual cells, many of which are polyploid with large mono- or binuclei, the SDZ reaches full development by GD 8. In addition to decidual cells, the SDZ also includes an increased number of small blood vessels as well as a range of immune cell types (i.e., large granular uNK, macrophage and dendritic cells) that are critical for early pregnancy establishment. By GD 8, the PDZ is markedly degenerated, and from this day onwards, placental and embryonic expansion progressively replaces the SDZ. Such an expansion transforms the antimesometrial decidua to a thin layer of cells termed the decidua capsularis. With pregnancy progression, the mesometrial decidua thins to the decidua basalis to accommodate the enlarging placenta, containing the embryonic-derived spongiotrophoblast cell layer and labyrinth zone. Blood vessels are denoted by BV. The dotted arrow indicates the direction of SDZ expansion. Elements of this artwork were adapted in modified form with permission from Lim and Wang (2010).

Following oocyte fertilization in the oviduct (or fallopian tube in the human), the single-celled zygote—through a series of cell divisions—develops to the advanced morula stage upon entry into the uterine cavity, a developmental journey of 3–4 and 5–7 days in the mouse and human, respectively (Carson et al., 2000; Wang and Dey, 2006; Evans et al., 2016). On the afternoon of gestation day (GD) 4 in the mouse, the late-stage blastocyst—comprising an inner cell mass (ICM) and an outer trophectodermal cell layer—attaches to the apical surface of the luminal epithelial lining of the implantation chamber (or crypt) of a transiently receptive endometrium (Figures 1A,B). Trophectodermal attachment to the anti-mesometrial side of the uterus is termed “the attachment reaction” in which the ICM of the blastocyst orients toward the mesometrial pole (the presumptive site for placentation) of an increasingly vascularized endometrium (Figures 1A,B).

In the human, the putative attachment reaction is considered to occur within the uterine fundus during the mid-secretory phase of the cycle (or ∼7–10 days after ovulation) (Evans et al., 2016). Unlike the mouse, the ICM of the apposed human blastocyst orientates toward the point of attachment during the implantation process. In both species, the temporal window of endometrial receptivity is relatively short (a ∼24 h implantation window for the ∼4–5 day murine estrous cycle, and a ∼2–4 day implantation window for the ∼28–30 day human menstrual cycle). Importantly, the opening of the endometrial receptivity window must synchronize with the on-time arrival of a competent blastocyst if successful implantation is to occur within this short time frame.

From GD 3 onwards in the mouse, a synchronous postovulatory rise in systemic progesterone levels is critical for the estrogen-primed endometrium to enter the transient receptive state that is permissive to embryo attachment, adhesion and ultimately invasion (Cha et al., 2012b; Namiki et al., 2018). Produced and secreted from newly formed corpora lutea of the ovary, progesterone primarily suppresses pre-ovulatory estrogen-induced proliferation of endometrial epithelia that occurred during the pre-receptive period (GD 1–3). The net result of this progesterone exposure is the transition of the endometrial epithelium from a proliferative to a differentiative state, a cellular transition that is conducive to blastocyst attachment. Detailed later, a majority of the molecular mechanisms identified to date that drive endometrial progesterone responses during this period have been shown to directly or indirectly suppress estrogen-induced epithelial proliferation. By GD 4, the underlying stroma synchronously proliferates in response to both progesterone and a small nidatory spike in estrogen, which together trigger the endometrial receptive state for embryo attachment.

On the afternoon of murine GD 5, subepithelial stromal cells encircling the nidating blastocyst within the epithelial implantation chamber undergo rapid progesterone-dependent proliferation. Toward the anti-mesometrial (AM) pole, proliferating stromal cells differentiate to form the primary decidual zone (PDZ). The transformation of stromal fibroblasts into densely packed epithelioid decidual cells of the PDZ is termed decidualization (Gellersen and Brosens, 2014; Figure 1B). By acting as a transient permeability barrier against immune cells and other potential harmful responses from the maternal compartment, the avascular PDZ is thought to protect the embryo following loss of the implantation chamber epithelium (Tung et al., 1986; Tan et al., 2002). For example, as a mechanism to avoid sudden oxidative stress, it’s proposed that the PDZ enables the gradual stromal acclimation of the embryo as it leaves the hypoxic epithelial environment of the implantation chamber to the relatively normoxic conditions of the stromal compartment (Tung et al., 1986). By GD 6, the PDZ is completely formed and is surrounded by a secondary decidual zone (SDZ; Figures 1B,C). At this time, the PDZ ceases cellular proliferation and begins to degenerate while the SDZ, enriched with small blood vessels, continues to proliferate. Fully developed at GD 8, the SDZ comprises large terminally differentiated decidual cells, many display polyploidy due to endoreduplication, which results in large mono- or binuclear cellular subpopulations. To meet the nutritional demands of a rapidly developing murine embryo, repeated rounds of DNA replication without cytokinesis is thought to be one of a number of strategies by which decidual cells rapidly increase histiotropic protein synthesis through enhancing transcriptional output (Das, 2009).

Decidualization spreads throughout the anti-mesometrial region forming the anti-mesometrial decidua before advancing to the mesometrial region, which eventually will form the decidua basalis (Figure 1C). After GD 8, the SDZ (now the anti-mesometrial decidua) is progressively replaced by placental and embryonic growth except for a thin cellular layer, termed the decidua capsularis (Figure 1C). Diametrically opposite, the mesometrial decidua gradually thins to the decidua basalis to accommodate formation and enlargement of the placenta (Figure 1C). In the case of the mouse, maintenance of pregnancy to parturition relies on sustained progesterone secretion from the corpus luteum. In the human, following the luteoplacental shift at 5–7 weeks of gestation (Turco and Moffett, 2019), the fetal placenta maintains continuously high levels of progesterone throughout the remaining two trimesters (Evans et al., 2016).

Unlike the mouse and the majority of placental mammals, in the human, along with anthropoid primates and a few non-primate species (i.e., the spiny mouse, the elephant shrew, and certain bat species), decidualization is not triggered by an implanting conceptus (Emera et al., 2012; Gellersen and Brosens, 2014; Evans et al., 2016; Bellofiore et al., 2017). Instead, decidualization only requires systemic hormones and local signals (in particular, cAMP) during the progesterone-dominant mid-secretory phase of a non-conception cycle (Gellersen and Brosens, 2003, 2014). Once initiated, progressive development and expansion of the endometrial decidual response is predicated on persistent progesterone exposure that only occurs if pregnancy is established and maintained (Evans et al., 2016). Fortunately for translational investigations, isolated human endometrial stromal cells can decidualize *in vitro*, requiring only cAMP, estradiol, and a progestin in the culture medium (Brosens et al., 1999). This cell assay has proven to be invaluable in translationally validating findings first made in the mouse.

### Decidualization: The Gateway to Placentation

While encapsulating the invading conceptus, decidual cells provide histiotropic nutrition until uteroplacental perfusion is established, which occurs after GD 8 in the mouse and by the end of the first trimester in the human (Cha et al., 2012b; Gellersen and Brosens, 2014; Evans et al., 2016; Ander et al., 2019; Turco and Moffett, 2019). Further underscoring their pleiotropic properties, decidual cells support an immunotolerant *milieu* to prevent rejection of the hemiallogeneic conceptus (Ander et al., 2019). As mentioned, decidual cells are also responsible for protecting against maternal stress stimuli (i.e., changes in oxygen tension and production of free radicals). The physical barrier presented by large decidual cells with their tight intercellular junctions serves as one strategy to regulate the orderly invasion of the conceptus into the endometrial compartment, which is critical not only for effective placenta formation but also to safeguard uterine integrity for future pregnancies (Emera et al., 2012). In the human at least, compelling data support decidual cells as biosensors that negatively select for non-viable embryos to prevent early establishment of pregnancies predestined for failure in the first or subsequent trimesters (Teklenburg et al., 2010; Brosens et al., 2014).

Apart from decidual cells, a large percentage of the decidua is comprised of a variety of maternal immune cells: uterine natural killer (uNK) cells [termed decidual NK (dNK) cells in the human (Sojka et al., 2019)], macrophages, T-regulatory cells and dendritic cells (Ander et al., 2019). Representing the majority of the maternal immune cells in the decidua, NK cells surrounding the maternal spiral arterioles are critical for promoting an immunosuppressive microenvironment for the invading hemiallogeneic conceptus (Fu et al., 2013). As a critical step toward placentation, NK cells also remodel spiral arterioles to enable endovascular trophoblast invasion, which leads to hemochorial placentation in the mouse and human (Evans et al., 2016). Further highlighting the functional versatility of these immune cells, NK cells limit excessive trophectodermal uterine invasion by eliciting apoptosis of the advancing cellular front of the extravillous trophectoderm of the conceptus (von Rango et al., 2003; Sliz et al., 2019). Data also support dNK cells in the clearance of premature senescent decidual cells (Brighton et al., 2017), a strategy by which immune cells maintain decidual health to avoid embryo implantation failure, early miscarriage, or pre-term birth (Cha et al., 2012a). Importantly, recent single cell profiling investigations have begun to identify the extracellular crosstalk signals (i.e., cytokine/cytokine receptor and other ligand/receptor pairs) that operate between the trophoblast, decidual, and immune cell-types (Nelson et al., 2016; Pavlicev et al., 2017; Suryawanshi et al., 2018; Vento-Tormo et al., 2018).

Collectively, investigations of uterine periimplantation biology demonstrate that progesterone-dependent endometrial receptivity and decidualization are crucial reproductive cellular processes that comprise a tightly synchronized continuum of endometrial cellular changes in which the successful conclusion of one cellular process relies on the successful completion of preceding cellular events (Cha et al., 2012b). Importantly, a number of adverse obstetric outcomes—early fetal miscarriage due to placental insufficiency, placenta accreta, preeclampsia, fetal growth restriction, and preterm birth—have been causally linked to placental abnormalities, the etiologic origins of which are suspected to arise due to defective decidualization (Norwitz et al., 2001).

As with any biology that can succumb to pathology, advancing our understanding of the critical molecular mechanisms that drive normal progesterone-dependent endometrial responses during early pregnancy is a prerequisite to developing novel mechanism-based diagnostics, prognostics and therapeutics not only for implantation failure but also for later gestational complications that occur due to derailment of normal endometrial receptivity and decidualization.

For the remainder of the review, a brief synopsis of the progesterone receptor (PGR) is provided before describing a signaling network that typifies the quintessential mediator role of the early progesterone response, which is required for development of the endometrial receptive and decidualized state. Finally, the review concludes by showcasing one example each of an epithelial and stromal modifier of the progesterone response that is critical for early pregnancy establishment.

## Transcriptional Reprogramming Underpins Endometrial Responses to Progesterone During the Periimplantation Period

### The Endometrial Progesterone Receptor: The Apex Transcriptional Regulator

The majority of tissue and cellular responses to systemic progesterone are mediated by the PGR, which is a group C member of nuclear receptor subfamily 3 (NR3C), a subfamily of 3-ketosteroid binding receptors of the nuclear receptor superfamily of transcription factors (Tsai and O’Malley, 1994; Mangelsdorf et al., 1995; DeMayo and Lydon, 2020). The PGR is modular in structure with defined functional domains, which include an extended N-terminus, a central DNA-binding motif with a hinge region, followed by the ligand-binding sequence; reviewed in Diep et al. (2015) and Grimm et al. (2016). Three activational domains are dispersed throughout the PGR, which interface with primary coregulators during transcriptional complex assembly. Not shared by most members of the superfamily, the PGR comprises two receptor isoforms (PGR-A and PGR-B) that are identical in sequence except that the human PGR-A isoform lacks the first 164 amino acid residues of the N-terminal domain. The absence of this amino acid sequence contributes, in part, to the different transactivational properties of PGR-A and PGR-B (Bain et al., 2000). Encoded by a single gene that uses the PGR-B distal and PGR-A proximal promoters to modulate expression of the corresponding isoform (Kastner et al., 1990), PGR-A and PGR-B are usually co-expressed in target tissues. As a result, the progesterone response of a target tissue is viewed as dependent on the relative ratio of the PGR-B and PGR-A isoforms within a cell population, in which discrete isoform homodimers and/or heterodimers mediate specific transactivational responses on target gene expression.

The mechanism of PGR action was originally viewed as progesterone hormone diffusing the plasma membrane of a target cell to bind the ligand-binding domain of the PGR; prior to ligand binding, PGR is pre-assembled within a multimeric protein complex (Pratt and Toft, 1997). Ligand binding elicits a conformational change in PGR [“the activation step”; O’Malley et al., 1969; O’Malley and Schrader, 1972] with concomitant disassembly of the complex. Following activation, ligand-bound PGR translocates to the nucleus to directly bind as a dimer (or monomer) to a specific progesterone response element(s) (PREs) within a target gene promoter or distant enhancer. The DNA-bound PGR along with coregulators (i.e., coactivators and corepressors) and ancillary cofactors assemble within the developing transcriptional complex to up- or down-regulate target gene expression, the ultimate molecular output of the progesterone response in a tissue. In the uterus, as in many target tissues, ovarian estrogen induces PGR expression, *via* its nuclear receptor [the estrogen receptor–α (ESR1); Graham and Clarke, 1997]. However, in many physiological contexts, estrogen-induced PGR-mediated signaling suppresses ESR1 activity, which is a critical regulatory response for uterine receptivity and implantation as detailed below.

Cell assays also reveal that post-translational modifications (PTMs) control PGR expression levels as well as the nuclear receptor’s transactivational activity, intracellular trafficking and target gene selection properties (Hagan and Lange, 2014). Moreover, *in vitro* studies show that PGR can indirectly modulate gene expression without the requirement for direct DNA binding (Grimm et al., 2016). The PGR has also been linked to non-genomic effects that occur outside the confines of the nucleus (Hagan and Lange, 2014; Grimm et al., 2016). Within an *in vitro* context, these mechanisms of action investigations provide provocative concepts to explain the possible diverse mechanisms by which PGR markedly expands its signaling capability beyond the classical mode of action. However, whether these non-classical modes of PGR signaling can be functionally linked to physiological and/or pathophysiological endpoints in the uterus has yet to be established.

Irrespective of whether any of these mechanisms are involved, early investigations on the PGR knockout (PRKO) mouse unequivocally confirmed the singular *in vivo* importance of the PGR transcription factor—and, by extension, the receptor’s downstream molecular targets (unknown at the time)—in female reproductive biology in general and in endometrial function in particular (Lydon et al., 1995). These investigations and others also confirmed that absence of PGR-mediated signaling results in endometrial unopposed estrogen action, which contributes to the PRKO infertility phenotype (Lydon et al., 1995) as well as endometrial pathologies, such as endometriosis (Fang et al., 2004). However, studies using PGR isoform specific knockout mice surprisingly revealed that only the PGR-A isoform is essential for pregnancy establishment, indicating PGR-B as well as isoform heterodimerization are dispensable for murine fecundity (Mulac-Jericevic et al., 2000, 2003). Importantly, however, it should be noted that both PGR isoforms are required for pregnancy success in the human (Kaya et al., 2015), highlighting a clear distinction between human and mouse.

Since its discovery as a nuclear receptor transcription factor (Conneely et al., 1986; Jeltsch et al., 1986), we have known that ligand-bound PGR, in coordination primarily with ESR1 signaling, must render a receptive and decidualized endometrial phenotype through control of transcriptional networks in a cell-type specific manner. Therefore, tremendous efforts have been expended toward identifying and characterizing the pivotal molecular signals that mediate and modify the endometrial progesterone response. Such efforts are driven by the hope that, with this deeper mechanistic understanding of endometrial PGR action, new molecular signals may be identified that contribute to more effective clinical diagnostics and therapeutics to address periimplantation failure as well as adverse pregnancy outcomes that manifest in later trimesters. Furthermore, such mechanism-based precision medicine could conceivably be repurposed to treat a variety of uterine pathologies resulting from aberrant progesterone responsiveness.

## Endometrial Molecular Mediators of the Progesterone Receptor During the Preiimplantation Period

Successful embryo implantation relies not only on bidirectional communication between the embryonic trophectoderm and endometrium but also on signaling crosstalk between the epithelial and stromal cellular compartments of the endometrium (Cha et al., 2012b; Hantak et al., 2014). The use of state-of-the-art engineered mouse models in tandem with high throughput transcript profiling methods—and more recently cistromic analysis—have identified signaling mediators and modifiers of endometrial PGR action, many of which belong to major paracrine signaling factor families that control the most fundamental cellular processes in pre-and post-natal tissue development and function.

### The Indian Hedgehog Signaling Axis

The Indian hedgehog (IHH) morphogen is a member of the highly conserved hedgehog family of ligands (Ng and Curran, 2011), which includes sonic and desert hedgehog. As diffusible morphogens, hedgehog family members control a wide-range of biological processes, including cellular proliferative and differentiative programs as well as short-range intercellular communication. Cellular processes that are crucial for coordinated development of the vertebrate body plan, organogenesis, tissue homeostasis, stem cell maintenance, and oncogenesis.

**FIGURE 2:**
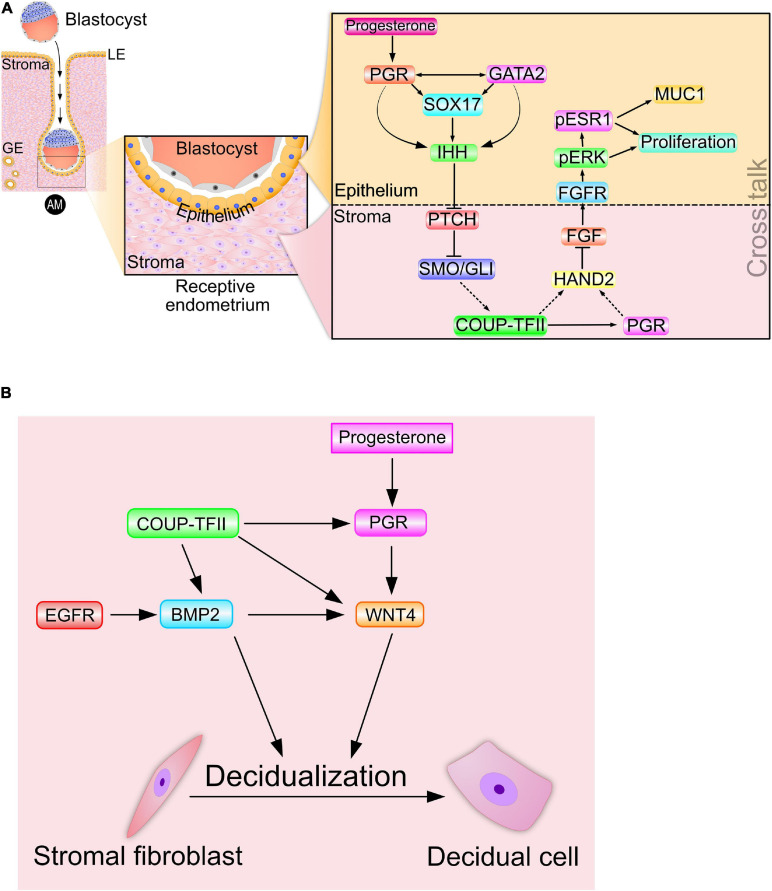
Signaling cross talk drives progesterone-dependent endometrial receptivity and decidualization. **(A)** Spanning the epithelial-stromal divide of the endometrium, the progesterone-PGR-IHH-COUP-TFII signaling axis primarily controls ESR1 activity in the epithelium. Suppression of ESR1 activity is a prerequisite for luminal epithelial differentiation of the endometrium to be receptive to embryo attachment and implantation. Note: Msx 1 and 2—and many other important mediator signals—that are important in this process are not shown. **(B)** A representative signaling network that is essential for progesterone driven endometrial stromal cell decidualization is shown. The dotted arrows indicate signaling relationships for which direct regulatory control has yet to be established. Adapted with permission in modified form from Wu et al. (2018).

Prior to murine embryo implantation, progesterone increases *Ihh* transcript levels in the luminal epithelium of the endometrium (Matsumoto et al., 2002; Takamoto et al., 2002). As described below, subsequent genome-wide chromatin occupancy studies would reveal that PGR directly binds the murine *Ihh* locus to mediate progesterone-induction of *Ihh* transcription directly (Wang et al., 2018). Interestingly, recent transcriptomic and cistromic analysis in conjunction with mouse studies reveal that the SOX17 transcription factor, a member of the SRY-determining region Y-related high-mobility group (HMG) box (SOX) family of transcription factors (Grimm et al., 2019), is both a direct target of PGR and required for full progesterone-induction of epithelial *Ihh* expression in the murine endometrium (Wang et al., 2018; Figure 2A). Described in more detail later, the GATA2 transcription factor also acts along with PGR and SOX17 to directly control *Ihh* expression in the mouse uterus. In the human, endometrial IHH levels are increased during the progesterone-dominant secretory phase of the menstrual cycle (Wei et al., 2010), supporting human IHH as a progesterone responsive target during the window of implantation. Further translational support for IHH as a PGR target arises from recent investigations using ulipristal acetate (UPA), a selective PGR modulator (SPRM) (Whitaker et al., 2017). Acting as a mixed progestin agonist and antagonist, UPA is known to exert well defined PGR modulator associated endometrial changes (PAEC). Indicated for women who are susceptible to heavy menstrual bleeding, UPA was shown to significantly increase endometrial *IHH* levels compared to peak levels observed in endometrial tissue biopsied from untreated women during the progesterone-dominant secretory phase of the cycle.

Studies using the PRKO mouse and a conditional *Ihh*-knockout mouse collectively demonstrated that progesterone induction of *Ihh* was through its nuclear receptor (Takamoto et al., 2002), and that elaboration of the receptive and decidualized endometrium relies on this induction (Lee et al., 2006). Importantly, examination of the pathohistology of the conditional *Ihh*-knockout endometrium revealed the presence of numerous cystic glandular ducts and a hyalinized stroma (Lee et al., 2006; Franco et al., 2010), histological hallmarks of a persistent estrogenized uterus. As a paracrine diffusible morphogen that facilitates endometrial epithelial-stromal crosstalk, epithelial-derived IHH was shown to activate the canonical hedgehog signal transduction pathway within the subluminal stroma (Figure 2A), thereby initiating epithelial-stromal crosstalk. The cognate hedgehog effector pathway in the stroma is comprised of the IHH receptor patched-1 (PTCH1), the activated intracellular transducer: smoothened (SMO) and the glioma-associated oncogene homolog (GLI) transcription factors (Figure 2A). Activation of the IHH signaling axis culminates with the activator form of GLI translocating to the nucleus to control transcriptional programming by directly binding target gene promoters.

Further mapping the IHH signaling pathway, the Tsai group demonstrated that activation of hedgehog signaling supports the expression of the orphan nuclear receptor: chicken ovalbumin upstream promoter transcription factor II [COUP-TFII; or nuclear receptor subfamily 2, group F, member 2 (NR2F2)] in the endometrial stroma (Takamoto et al., 2002; Figure 2A). A pivotal differentiation factor in the mesenchyme, COUP-TFII controls a plethora of developmental and physiological processes, ranging from organogenesis, angiogenesis and inflammation to cell adhesion and cell fate specification (Wu et al., 2016). Infertility resulting from conditional ablation of *Coup-tfII* in the female mouse is due to failures in embryo implantation and stromal decidualization (Kurihara et al., 2007; Lee et al., 2010); endometrial defects that are associated with a heightened estrogenized uterus due to persistent increased ESR1 activity. In the human, abnormal reduction in both IHH and COUP-TFII levels in the endometrium is associated with endometriosis (Whitaker et al., 2017). Apart from maintaining prolonged ESR1 activity and stability, suppression of COUP-TFII levels is linked to increased proinflammatory cytokine levels (Li et al., 2013; Lin et al., 2014), angiogenesis (Fu et al., 2018), and local estrogen synthesis (Zeitoun et al., 1999; Zeitoun and Bulun, 1999; Attar et al., 2009), all of which markedly promote the pathogenesis of endometriosis.

Increased COUP-TFII expression by PGR-IHH signaling not only results in increased stromal PGR levels but also the levels of stromal heart and neural crest derivatives expressed transcript 2 (*Hand2*) (Li et al., 2011; Marinic et al., 2021; Figure 2A), which is a basic helix-loop-helix transcription factor. To date, studies have not established whether the COUP-TFII transcription factor directly or indirectly (i.e., *via* stromal PGR) increases stromal *Hand2* levels; however, HAND2 suppresses stromal fibroblast growth factor (*Fgf*) family members (*Fgf*−1, −2, −9, and −18) in the endometrium (Li et al., 2011; Figure 2A). Accordingly, conditional *Hand2* knockout mouse studies revealed that significant induction of stromal *Fgf* expression in the endometrium represents one of the molecular consequences of *Hand2* ablation (Li et al., 2011).

Through paracrine signaling within the normal endometrium, stromal FGFs bind their cognate transmembrane tyrosine kinase receptors (FGFRs with ancillary docking factors) located in epithelial cells. Engagement of stromal-derived FGF ligand with its epithelial-derived receptor elicits phosphorylation (and activation) of extracellular signal regulated kinases 1 and 2 (ERK1/2). In turn, activated ERK1/2 phosphorylate ESR1 to activate and stabilize ESR1 in the epithelium (Figure 2A). During the pre-receptive period, activated ESR1 not only triggers epithelial proliferation but also maintains expression of mucin 1 (MUC1) (Li et al., 2011). The apical surface of luminal epithelial cells expresses the MUC1 glycoprotein as a barrier to embryo attachment (Surveyor et al., 1995). Moreover, protracted proliferation of the glandular epithelium blocks expression of the leukemia inhibitory factor [LIF; Stewart et al., 1992), an interleukin 6 family cytokine member] as well as the Forkhead box A2 (FOX A2) transcription factor (Kelleher et al., 2017), which are both essential signals for embryo implantation. Noteworthy is the increasing number of clinical reports that implicate perturbation of HAND2 and FGF levels with aberrant progesterone responses in the human endometrium that lead to endometriosis, endometrial hyperplasia and carcinoma (Jones et al., 2013; Buell-Gutbrod et al., 2015; Logan et al., 2018; Kato et al., 2019).

Therefore, perturbation in the normal levels of any component of the progesterone-PGR-IHH-COUP-TFII-HAND2 signaling axis is predicted to result in unwarranted activation of epithelial ESR1 (Figure 2A).

### Endometrial Receptivity and Decidualization: Molecular Ties That Bind

Disclosing the intricate signaling networks that mediate progesterone-dependent epithelial-stromal crosstalk and drive endometrial receptivity also revealed the degree to which endometrial receptivity and decidualization are closely connected at the molecular level. Induction of COUP-TFII by the PGR-IHH signaling axis also increases levels of stromal bone morphogenetic protein 2 (*Bmp2*) (Kurihara et al., 2007), which is a member of the transforming growth factor beta (TGFβ) superfamily of cytokines. Increased *Bmp2* levels are associated with increased levels of wingless-type MMTV integration site (WNT) family member 4 (*Wnt 4*) in the stroma (Li et al., 2007; Figure 2B). Both clinical and mouse studies show that BMP2 and WNT 4 (along with COUP-TFII and IHH) are indispensable for PGR-dependent endometrial stromal cell decidualization (Takamoto et al., 2002; Kurihara et al., 2007; Lee et al., 2007; Franco et al., 2010). Also, epidermal growth factor receptor (*Egfr*) signaling is linked to increased levels of *Bmp2* and *Wnt4* in the murine uterus during early pregnancy (Large et al., 2014). Increasing this regulatory complexity to the next level are the findings that BMP2 induces members of the muscle segment homeobox (*Msx*) family of transcription factors (*Msx 1* and *2*) (Nallasamy et al., 2019), which also contribute to the epithelial-stromal crosstalk that is essential for endometrial receptivity (Daikoku et al., 2011; Nallasamy et al., 2012).

## Modifiers of PGR Mediated Responses in the Endometrial Epithelium and Stroma

Molecular modifiers of PGR activity modify its regulation of target gene transcriptional output through various mechanisms: from controlling PGR stability and ligand binding affinity to coregulating PGR-dependent gene expression programs. An increasing number of epithelial and stromal modifiers of PGR action has recently been characterized in the mouse, many of which have strong translational significance.

### The GATA 2 Transcription Factor: An Epithelial PGR Modifier

Binding an evolutionarily conserved short *GATA* DNA sequence, the six member family of *GATA*-binding transcription factors control a broad spectrum of biological processes, ranging from hematopoiesis, adipocyte development to pituitary gland function (Tremblay et al., 2018). Through DNA binding, GATA transcription factors primarily perform as “pioneer factors” by opening heterochromatin following the recruitment and assistance of epigenetic modifiers. With open chromatin, transcriptional regulators (i.e., coactivators and corepressors) combinatorially assemble at proximal promoters or distal enhancer elements of target genes to control transcriptional output.

Initial studies demonstrated a strong correlation between *GATA2* and *PGR* expression in human endometrial tissues (Rubel et al., 2012, 2016), suggesting a signaling connection. While GATA2 is expressed in the epithelial and stromal compartments of the murine endometrium, its expression is coincident with PGR expression in the endometrial epithelium (Rubel et al., 2012). For both transcription factors, epithelial expression levels peak just before entry into the window of receptivity (Rubel et al., 2012), only to rapidly decline thereafter. Engineered mice in which the function of GATA2 or PGR is selectively abrogated in the endometrial epithelium exhibit similar impairments in uterine receptivity and decidualization (Franco et al., 2012; Rubel et al., 2016), providing *in vivo* support for a mechanistic link between the two transcription factors.

**FIGURE 3:**
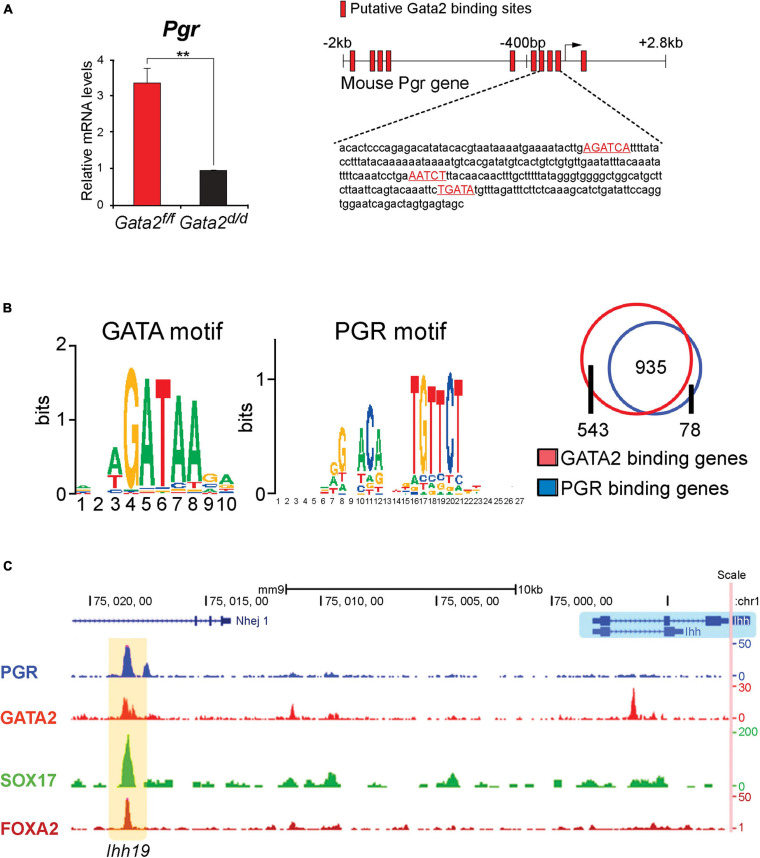
The GATA2 transcription factor is a direct epithelial modifier of PGR-mediated transcription in the murine endometrium. **(A)** Quantitative real-time PCR analysis reveals that absence of GATA2 in the endometrium of the ovariectomized mouse results in a significant reduction in *Pgr* transcript levels. Note: in the uterus of an ovariectomized control mouse, the majority of *Pgr* expression in located in the epithelial compartment (Rubel et al., 2016). Also note that the GATA2 transcription factor is conditionally ablated in PGR positive cells of the uterus in the *Gata2^*d*/d^* mouse. *In silico* analysis highlights numerous candidate DNA binding motifs for GATA2, which are located throughout the 5′ regulatory regions of the murine *Pgr* gene (red boxes) and red highlighted sequences. Chromatin immunoprecipitation and *in vitro* transient transfection experiments confirmed that many of these binding sites directly bind GATA2 and are functional (Rubel et al., 2016). **(B)** Using uterine tissue from ovariectomized mice treated with progesterone for 6 h, chromatin immunoprecipitation followed by genome-wide sequencing (ChIP-seq) identified enriched binding motifs for the GATA2 and PGR transcription factors within GATA2 binding intervals throughout the genome. The Venn diagram displays the progesterone responsive genes in the mouse uterus that contain binding sites for PGR or GATA2 within ± 25 kb of gene boundaries. Note: the significant overlap (935 genes) that represent genes jointly bound by PGR and GATA2 (Rubel et al., 2016). **(C)** The traces show the co-occupancy locations of the PGR, GATA 2, SOX17, and FOXA2 transcription factors at a distal enhancer region on the murine *Ihh* gene (Wang et al., 2018). Note: SOX17 is also a direct target of GATA2 and PGR (Wang et al., 2018); the FOXA2 transcription factor (not covered in this review) is critical for uterine glandular epithelial function that is required for pregnancy establishment (Kelleher et al., 2017). With permission, parts of this schematic were reproduced in modified form from Rubel et al. (2016) and Wang et al. (2018). **p < 0.01.

Importantly, examination of the murine endometrium with a conditional *Gata2* null mutation revealed that the levels of epithelial *Pgr* are significantly reduced when compared to control mice (Figure 3A; Rubel et al., 2016). These results indicated that endometrial GATA2 directly or indirectly controls the epithelial levels of PGR—and by extension, its target genes. Chromatin immunoprecipitation experiments demonstrated that GATA2 directly binds within the 5′ regulatory region (including the proximal promoter) of the murine *Pgr* gene (Figure 3B; Rubel et al., 2016), providing strong molecular support for GATA2 as a direct modifier of PGR expression. Interestingly, these studies also showed that GATA2 co-occupies with PGR, SOX17, and FOXA2 within regulatory regions that control *Ihh* expression (Figure 3C). Noteworthy, *Sox17* is also a direct target of GATA2 and PGR (Wang et al., 2018), and the FOXA2 transcription factor is required for glandular epithelial functions required for pregnancy establishment (Kelleher et al., 2017). These findings collectively suggest that GATA2 modifies progesterone signaling not only through direct regulation of *Pgr* transcription but also by jointly acting with PGR to regulate target gene expression (Rubel et al., 2016).

Of translational significance, integration of expression array datasets from human and murine endometrial tissue uncovered an evolutionarily conserved GATA2-PGR-SOX17 dependent regulatory network that is predicted to control the majority of endometrial target mediators of normal progesterone responsiveness (Rubel et al., 2016; Wang et al., 2018). Based on these observations, perturbation in endometrial GATA2 levels is predicted to cause endometrial pathologies due to progesterone resistance. Indeed, one report describes endometrial GATA2 levels are markedly decreased in women with endometriosis (Dyson et al., 2014). While in the mouse, ablation of GATA2 function in the endometrium results in epithelial hallmarks of unopposed estrogen signaling, which include abnormal epithelial integrity, characterized by extensive basal cell stratification (Franco et al., 2012; Rubel et al., 2016).

Interestingly, prostate studies reveal that GATA2 can bind upstream regulatory elements to increase expression levels of the androgen receptor [(AR) a NR3C subfamily member and close relative of PGR; Wu et al., 2014]. As a pioneer factor in prostate cells, GATA2 promotes chromatin accessibility at enhancer regions through recruitment of p300 histone acetyltransferase to generate active chromatin by acetylating lysine 27 in histone 3 (H3K27). Moreover, GATA2 has been shown to create and maintain regulatory chromatin loops between AR-bound distal enhancers and promoters of AR target genes by recruiting the mediator coregulator complex. Interestingly, a similar DNA looping mechanism has been put forward to explain the joint regulation by PGR and GATA2 of target genes in a murine mammary gland adenocarcinoma cell line (Magklara and Smith, 2009). Whether similar modifier mechanisms in the endometrial epithelium apply to GATA2 modification of *Pgr* expression levels and its signaling program warrants further investigation.

### Steroid Receptor Coactivator-2: A Stromal Modifier

**FIGURE 4:**
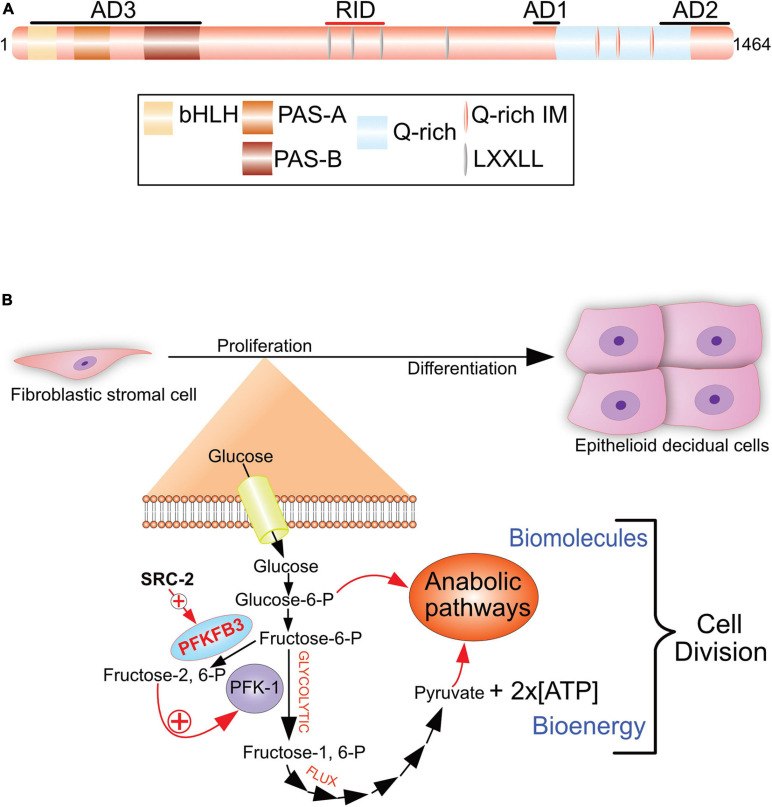
The SRC-2 coactivator is required for progesterone-dependent acceleration of the glycolytic flux that drives endometrial stromal cell decidualization. **(A)** The pleiotropic properties of SRC-2 are based on its complex protein functional domain organization. Activation domains 1–3 (AD1-3), receptor interaction domain (RID), basic helix-loop-helix domain (bHLH), the Per/ARNT/Sim domains -A and -B (PAS-A and –B), leucine-X-X-leucine-leucine [X denotes any amino acid (LXXLL)], and the glutamine-rich/interaction motif (Q-rich/IM) are indicated. **(B)** To generate sufficient numbers of epithelioid decidual cells to support embryo implantation, stromal fibroblasts of the endometrium rapidly proliferate in response to progesterone prior to their differentiation. By markedly increasing the rate of glucose uptake and glycolysis, an endometrial stromal cell rapidly produces two daughter cells following mitosis. Glycolysis from glucose to pyruvate is referred to as the glycolytic flux. Increasing the glycolytic flux serves to rapidly provide the necessary bioenergy and biomolecules to meet the urgent demands of a proliferating endometrial stromal cell that is about to form two daughter cells. The progesterone-dependent acceleration of the glycolytic flux requires SRC-2 co-regulation of PGR-mediated induction of PFKFB3 (Kommagani et al., 2013), a potent positive regulator of the glycolytic flux. Using its kinase domain, PFKFB3 converts fructose-6-P to fructose-2, 6-P, which allosterically activates PFK-1, a pivotal checkpoint of glycolysis. Increasing the glycolytic flux results in a net gain of two ATP molecules per glucose molecule catabolized, and the generation of glycolytic intermediates (i.e., glucose-6-P and pyruvate) to furnish the required precursors for macromolecular and organelle biosynthesis by downstream anabolic pathways. 6-Phosphofructo-2-kinase/fructose-2, 6-bisphosphatase 3, phosphofructokinase-1 are abbreviated by PFKFB3 and PF-1, respectively. With permission, aspects of this figure were reproduced in modified form from Szwarc et al. (2014b).

Steroid Receptor Coactivator-2 (SRC-2; also known as NCOA2, GRIP1, and TIF2) is a member of the p160/SRC family of coactivators, which also comprises SRC-1 and SRC-3 (Xu et al., 2009; Szwarc et al., 2014b). As pleiotropic coregulators, SRC family members regulate a myriad of physiological responses and clinicopathologies. The pleiotropic properties of SRCs are due to their large size and structural complexity, which enable this coregulator family to modulate both nuclear receptor and non-nuclear receptor signaling. Using the PGR as a molecular bait in a two-hybrid system, the first SRC family member (SRC-1) was isolated and characterized as a primary coactivator of DNA-bound nuclear receptors (Onate et al., 1995). Since then, SRC family members have been shown to act as non-overlapping coactivators of numerous members of the nuclear receptor superfamily (Xu et al., 2009). For example, early studies demonstrated a potent coactivator role for SRC-2 in PGR-mediated transactivation *in vitro* (Hofman et al., 2002). Numerous investigations demonstrated that SRCs transmit the activation signal from DNA-bound nuclear receptors to secondary coregulators (p300) and ancillary factors, which in turn stream the signal to the general transcriptional complex to induce target gene transcription (Xu et al., 2009). Although important for nuclear receptor function, the complex functional domain organization of SRCs—containing numerous composite protein-protein interaction surfaces (Figure 4A)—strongly predicted that this coregulator class may serve as multifunctional integrators for a broad array of signals distinct from those mediated by nuclear receptors.

Studies on the SRC-2 knockout (SRC-2KO) mouse were the first to demonstrate a critical role for this coactivator in both male and female reproductive function (Gehin et al., 2002). In the male, SRC-2 acts as an AR coactivator in the prostate and testes (Gehin et al., 2002; Mark et al., 2004; Ye et al., 2005; Vija et al., 2013). In the case of the SRC-2KO female, initial phenotype analysis indicated that the infertility phenotype arises due to significant placental hypoplasia (Gehin et al., 2002). However, selective ablation of SRC-2 in cells expressing the PGR revealed that endometrial SRC-2 is required earlier in gestation, specifically during the peri-implantation period (Mukherjee et al., 2006b). In this conditional knockout model, embryos fail to implant due to impaired progesterone-dependent endometrial stromal cell decidualization. In addition to gene ablation strategies, experimentally increasing SRC-2 levels in the murine uterus also leads to a severe subfertility phenotype, which is associated with impaired endometrial decidualization with enhanced estrogen sensitivity (Szwarc et al., 2014a). While SRC-2 levels in the epithelium and stroma of the endometrium do not change significantly during the human cycle or in the early pregnant mouse (Jeong et al., 2005; Mukherjee et al., 2006a), the above mouse studies underscore the critical importance of tight control of the levels of this coregulator to ensure normal endometrial function; note: perturbation in SRC levels is a frequent etiologic factor in many tissue pathologies (Xu et al., 2009).

Of translational importance, SRC-2 is also required for progesterone-driven decidualization of primary human endometrial stromal cells in culture (Kommagani et al., 2013), furnishing strong evolutionary support for this endometrial coregulator in periimplantation biology. Both human cell and mouse studies demonstrated that SRC-2 is indispensable for rapid progesterone-driven endometrial stromal cell proliferation, which is crucial for timely development of the receptive endometrium (Kommagani et al., 2013). Requiring markedly increased metabolic expenditure, this cell-division period accelerates enlargement of the endometrial stromal cell pool prior to its terminal differentiation into decidual cells (Sroga et al., 2012).

In accordance with its coregulator role in metabolism (Stashi et al., 2014; O’Malley, 2020), metabolomics revealed SRC-2 to be essential for progesterone-dependent acceleration of the glycolytic flux in cultured primary human endometrial stromal cells prior to their decidualization (Kommagani et al., 2013). Accelerating the glycolytic flux facilitates rapid supply of the necessary ATP levels and glycolytic intermediates to downstream anabolic pathways that produce biomass required for cell growth prior to mitosis (Vander Heiden et al., 2009). Specifically, SRC-2 is required for progesterone-induction of 6-phosphofructo-2-kinase/fructose-2, 6-bisphosphatase 3 (PFKFB3; Figure 4B), a bifunctional enzyme that is indispensable for a myriad of cellular processes from embryogenesis, post-natal cellular proliferation to cancer progression (Chesney et al., 2005; Chesney, 2006; Clem et al., 2008). Notably, the PFKFB3 enzyme was first discovered in the human placenta (Fukasawa et al., 2004) and induced by progestins in cultured human breast cancer cells (Novellasdemunt et al., 2012). The kinase function of PFKFB3 phosphorylates fructose-6-phosphate to fructose-2, 6-phosphate (Figure 4B). Fructose-2, 6-phosphate is a key allosteric activator of phosphofructokinase-1 (PFK-1), a critical rate-limiting checkpoint of the glycolysis pathway (Uyeda et al., 1981; Van Schaftingen et al., 1981). With acceleration of the glycolytic flux *via* the PFK-1 checkpoint, anabolic pathways (i.e., the pentose phosphate pathway) can accelerate endometrial stromal cell proliferation to ensure timely and complete decidualization. Both in cultured human endometrial stromal cells and in the mouse, inhibiting PFKFB3 activity blocks decidualization (Kommagani et al., 2013), underscoring the importance of this glycolytic regulator to the decidualization process.

While highlighting a critical modifier role for endometrial stromal SRC-2 in progesterone-dependent early pregnancy establishment, findings from these investigations pose fascinating questions for future studies: (1) Does stromal SRC-2 control other metabolic programs that promote endometrial stromal cell decidualization? The question is raised because SRC-2 (along with other SRC members) is an established coregulator of carbohydrate, lipid, and amino acid metabolism in other physiological systems (Stashi et al., 2014; O’Malley, 2020). Furthermore, SRC-2 in human endometrial cancer cells was recently shown to be essential for the normal performance of the pentose phosphate pathway in addition to maintaining cellular glycolytic capacity and oxidative phosphorylation (Szwarc et al., 2018b). As an anabolic multi-enzyme pathway, the pentose phosphate pathway generates NADPH for reductive biosynthesis as well as pentoses (including ribose 5-phosphate) to generate nucleotides for DNA and RNA synthesis. Together, these SRC-2 dependent pathways are required to drive rapid proliferation and anchorage independent growth of this cancer cell type (Szwarc et al., 2018b). Whether SRC-2 is also required for pentose phosphate pathway functionality in normal human endometrial cells awaits investigation; (2) Is impaired control of the glycolytic pathway an underpinning for the implicated role of SRC-2 in endometrial pathologies as observed in patients with polycystic ovary syndrome or endometrial cancer? A majority of cancers rely on the induction of PFKFB3 and the acceleration of the glycolytic flux for enhanced cellular proliferation (Clem et al., 2008; Yalcin et al., 2009; Novellasdemunt et al., 2013). Increased expression levels of SRC-2 (and SRC-3) are detected in endometrial hyperplasia as well as in the endometrium of patients diagnosed with polycystic ovary syndrome (Gregory et al., 2002), a patient group susceptible to endometrial cancer (Coulam et al., 1983; Pillay et al., 2006). Indeed, increasing SRC-2 levels in the murine endometrium results in endometrial hyperplasia, further supporting the above translational observations (Szwarc et al., 2014a). Therefore, it will be interesting to determine whether perturbation in SRC-2 levels causes abnormal induction of PFKFB3 that promotes these endometrial proliferative disorders. As inhibitors of PFKFB3 show recent promise as plausible treatment options for proliferative disorders such as cancer (Wang Y. et al., 2020), targeting PFKFB3 may also be an option to treat endometrial pathologies with an unchecked proliferative phenotype; (3) Does epithelial-derived SRC-2 have a role in endometrial receptivity and decidualization? Similar to GATA2, SRC-2 is expressed in both the epithelial and stromal compartments of the human and mouse endometrium (Jeong et al., 2005; Mukherjee et al., 2006a). As described above, stromal-derived SRC-2 has a clear modifier role in progesterone signaling processes in the endometrium that are for important embryo implantation; however, the involvement of endometrial epithelial SRC-2 in the implantation process is unknown; and finally (4) In addition to metabolic pathways, can SRC-2 control other progesterone responsive signals that are crucial for endometrial stromal cell decidualization? Recent transcriptomic and cistromic investigations have not only underscored the critical importance of SRC-2 in the full induction of the majority of known molecular mediators of progesterone during human endometrial stromal cell decidualization but have also uncovered new gene targets coregulated by SRC-2 and the PGR that may represent new lines of future investigation to understand coregulator control of early progesterone responses in the endometrium during the periimplantation period (Szwarc et al., 2018a).

## Conclusion and Future Perspectives

Even after 75 million years of evolutionary divergence (Nei and Glazko, 2002), the uterus of the human and mouse still share a remarkable degree of cellular and molecular conservation. This conservation has made the mouse the *de facto* model for interrogating endometrial function in a living eutherian mammal. Due to its genetic and experimental malleability, the mouse has shed new light into the “black box” of endometrial periimplantation biology (Macklon et al., 2002), particularly relating to endometrial progesterone responses before placentation. Recent molecular phenotyping approaches, transcriptomics and cistromics in particular, applied to the endometrium of the engineered mouse have enabled a more integrative analysis of endometrial gene expression and regulatory networks on a genome-wide scale. From these studies, we can now appreciate the tremendous regulatory complexity required to mediate and modify endometrial progesterone responses during the periimplantation period. Such complexity underpins endometrial epithelial-stromal cross talk that drives pregnancy establishment. Further underscoring this regulatory complexity is the myriad of signals that are involved, which include transcription factors, growth factors, morphogens and cytokines as well as metabolic signals. Because of the immense scale and complexity of these interconnected signaling networks, codification of the regulatory hierarchical mechanisms by which these networks are coordinately integrated in a spatiotemporal manner will be a formidable but important challenge.

This review profiled just a small selection of endometrial molecular mediators and modifiers of the progesterone response that are essential for embryo implantation. While many of these factors are causally linked to the development of the decidual cell from the stromal fibroblast, their role in the manifold functions of the decidual cell following its development is not fully understood. As described previously, many of these functions include angiogenesis, local immunosuppression, immune cell influx and prevention of pre-mature senescence. Exploration of these aspects of decidual cell function in the mouse using cre-dependent gene ablation technologies can only occur with the development of new cre drivers that are selectively expressed in the stromal cell following decidualization.

With the advent of CRISPR/Cas9 gene editing methodologies (Doudna and Charpentier, 2014), structure-function analysis of the molecular mediators and modifiers of the endometrial progesterone response is now a realizable goal in the mouse. With this technology, the precise mechanistic dissection of functional domains, critical protein-protein interaction motifs and PTM sites of a mediator or modifier of progesterone-driven endometrial receptivity and decidualization will be possible. Already cis-regulatory elements of transcription factors have been functionally evaluated in the mouse by CRISPR/Cas9 methods (Wang et al., 2018). With our ability to map the genome-wide locations of distal enhancer elements of transcription factors and coactivators to their potential target gene promoters using such 3D genomic technologies as high throughput chromosome conformation capture (Hi-C) (Dekker et al., 2013), elucidation of the individual or combined *in vivo* importance of these enhancer-promoter pairs to murine endometrial periimplantation biology is now a reality.

Single cell or cell atlas technologies are recently responsible for stunning advances in our molecular understanding of the interactome that operates between cells derived from the human trophoblast and the endometrial stroma (Pavlicev et al., 2017; Rajagopalan and Long, 2018; Suryawanshi et al., 2018; Vento-Tormo et al., 2018). Elegant single-cell RNA sequencing (scRNAseq) investigations of the normal human endometrium have also resolved its cellular heterogeneity into multiple dimensions across the menstrual cycle (Giudice, 2020; Wang W. et al., 2020). Such studies have provided novel molecular descriptors for menstrual-cycle phase transitions, biomarkers that signal the emergence of the “window of implantation” (WOI), and critical insights into the spatiotemporal dynamics of intercellular communication systems that modulate with the changing hormonal environment of the cycle. In many of these studies, single cell transcriptomics resolved previously unknown rare cell types from supposedly homogenous cell populations, a capability that is lacking with conventional tissue-level or bulk transcriptomics (Pavlicev et al., 2017; Rajagopalan and Long, 2018; Suryawanshi et al., 2018; Vento-Tormo et al., 2018; Fitzgerald et al., 2019; Cochrane et al., 2020; Giudice, 2020; Lucas et al., 2020; Wang W. et al., 2020). Just as single-cell approaches have significantly informed our understanding of various interactomes that coexist in human endometrial cell populations so too have these methods recently advanced our knowledge of interactomes in the mouse uterus, particularly within the endometrial epithelium and placenta (Nelson et al., 2016; Wu et al., 2017). These studies along with the future application of these approaches and emergent derivations thereof [i.e., high definition spatial transcriptomics as well as scRNAseq combined with ATAC-seq methods (Hendrickson et al., 2018; Vickovic et al., 2019)] to the murine endometrium of the engineered mouse will not only complement but will markedly extend recent findings made with human endometrial cells.

At present there is no *in vivo* model that matches the mouse in terms of interrogating the molecular mechanisms by which progesterone governs endometrial function at the whole organism level. Therefore, the engineered mouse will continue to enrich our understanding of endometrial progesterone responsiveness as it relates to pregnancy establishment. Looking forward, we predict that molecular phenotyping of the murine endometrium at high resolution—in concert with translational studies—will provide a more comprehensive molecular understanding of progesterone’s role not only in endometrial receptivity, decidualization and implantation success but also in gynecological morbidities suffered by women who are diagnosed with an endometrium with a compromised progesterone response.

Far from hype, the aforementioned provides genuine hope that we are entering a propitious time for the accelerated development of personalized mechanism-based diagnostic and targeted therapeutics for endometrium-based infertility and associated progesterone-responsive disorders. The ultimate hope is that such developments will be actionable in the clinic sooner rather than later to maintain, restore or optimize endometrial function in otherwise healthy women.

## Author Contributions

All authors contributed equally to researching, writing, and reviewing the manuscript.

## Conflict of Interest

The authors declare that the research was conducted in the absence of any commercial or financial relationships that could be construed as a potential conflict of interest.
